# Prevalence of preoperative anemia, abnormal mean corpuscular volume and red cell distribution width among surgical patients in Singapore, and their influence on one year mortality

**DOI:** 10.1371/journal.pone.0182543

**Published:** 2017-08-04

**Authors:** Yilin Eileen Sim, Hide Elfrida Wee, Ai Leen Ang, Niresh Ranjakunalan, Biauw Chi Ong, Hairil Rizal Abdullah

**Affiliations:** 1 Department of Anaesthesiology, Singapore General Hospital, Singapore, Singapore; 2 Department of Occupational Medicine, Singapore General Hospital, Singapore, Singapore; 3 Department of Haematology, Singapore General Hospital, Singapore, Singapore; 4 Yong Loo Lin School of Medicine, National University of Singapore, Singapore, Singapore; Universidade Nova de Lisboa Instituto de Higiene e Medicina Tropical, PORTUGAL

## Abstract

**Introduction:**

Preoperative anemia and high red cell distribution width (RDW) are associated with higher perioperative mortality. Conditions with high RDW levels can be categorized by mean corpuscular volume (MCV). The relationship between RDW, anemia and MCV may explain causality between high RDW levels and outcomes. We aim to establish the prevalence of preoperative anemia and distribution of RDW and MCV among pre-surgical patients in Singapore. In addition, we aim to investigate the association between preoperative anemia, RDW and MCV levels with one-year mortality after surgery.

**Methods:**

Retrospective review of 97,443 patients aged > = 18 years who underwent cardiac and non-cardiac surgeries under anesthesia between January 2012 and October 2016. Patient demographics, comorbidities, priority of surgery, surgical risk classification, perioperative transfusion, preoperative hemoglobin, RDW, MCV were collected. WHO anemia classification was used. High RDW was defined as >15.7%. Multivariate regression analyses were done to identify independent risk factors for mild or moderate/severe anemia and high RDW (>15.7). Multivariate cox regression analysis was done to determine the effect of preoperative anemia, abnormal RDW and MCV values on 1-year mortality.

**Results:**

Our cohort comprised of 94.7% non-cardiac and 5.3% cardiac surgeries. 88.7% of patients achieved 1 year follow-up. Anemia prevalence was 27.8%—mild anemia 15.3%, moderate anemia 12.0% and severe anemia 0.5%. One-year mortality was 3.5%. Anemia increased with age in males, while in females, anemia was more prevalent between 18–49 years and > = 70 years. Most anemics were normocytic. Normocytosis and macrocytosis increased with age, while microcytosis decreased with age. Older age, male gender, higher ASA-PS score, anemia (mild- aHR 1.98; moderate/severe aHR 2.86), macrocytosis (aHR 1.47), high RDW (aHR 2.34), moderate-high risk surgery and emergency surgery were associated with higher hazard ratios of one-year mortality.

**Discussion:**

Preoperative anemia is common. Anemia, macrocytosis and high RDW increases one year mortality.

## Introduction

Preoperative anemia is associated with an increased risk of perioperative transfusion and adverse outcomes after surgery, including morbidity and mortality [1–3]. Patient blood management (PBM) programs which aim to optimize patients’ anaemia status preoperatively are fast becoming the international standard of care. PBM implementation has reduced perioperative transfusion[4,5], hospital length of stay and readmission rates after elective surgeries[6]. Thus, there is a compelling need to screen for anemia in the preoperative patient population. The prevalence of anemia in the population varies depending on the age, gender, [7–9] comorbidities and type of surgery that they are presenting for [10]. However, data on the pre-surgical population in Singapore is currently lacking as most studies on preoperative anemia were done in the western population. As previous work on global anemia burden has shown, there is a regional variation in anemia prevalence [11]. Hence, we postulate that Singapore may have different anemia prevalence and post-operative outcomes from Western countries due to the different racial mix, dietary profile, endemic disease prevalence as well as epigenetic influence.

Red cell indices such as the red cell distribution width (RDW) and mean corpuscular volume (MCV) are routinely performed in the automated full blood count. MCV is used to classify cell morphology into macrocytic, microcytic or normocytic, while RDW is a measure of anisocytosis, reflecting the variation in red blood cell sizes. [12,13]. Recently, high RDW levels are an independent marker for mortality, even after adjusting for anemia, both in the community [14–16] and perioperative setting. High preoperative RDW levels is associated with reduced survival after colorectal cancer surgery, in children undergoing surgical repair of congenital heart disease, and in elderly patients who underwent fixation of hip fracture.[17–20] It has been postulated that higher RDW levels reflects abnormal bone marrow activity, which may be due to chronic malnutrition, inflammation or high levels of circulating reactive oxygen species. [21–25] High RDW levels are found in a heterogeneous group of conditions, which can be further categorized by the MCV levels [26]. Currently most studies on RDW adjust for anemia, but few examine its relationship with MCV which may help differentiate between the causes of high RDW levels, and explain the causal relationship between high RDW levels and poorer outcomes.

This study aims to establish the prevalence of preoperative anemia and distribution of RDW and MCV based on age, gender and comorbidities among patients undergoing surgery in Singapore. Furthermore, we aim to investigate the association between preoperative anemia, RDW and MCV levels on one-year mortality after surgery. These information could help to direct screening efforts for preoperative anemia as part of the PBM implementation strategy and contribute to existing knowledge on perioperative risk stratification based on anemia, RDW and MCV.

## Methodology

### IRB approval

Institutional Review Board approval was obtained (Singhealth CIRB 2014/651/D) prior to the commencement of the study, which waived the requirement for individual informed consent. We retrospectively analyzed the electronic medical records of 98,685 patients aged 18 and older who underwent surgery under general or regional anesthesia between 1 January 2012 and 31 October 2016 in Singapore General Hospital, a 1700-bedded tertiary academic hospital in Singapore. These clinical records were sourced from our institution's clinical information system (Sunrise Clinical Manager (SCM), Allscripts, IL, USA) and stored in our enterprise data repository and analytics system (SingHealth-IHiS Electronic Health Intelligence System—eHINTS). Mortality data in the system was synchronized with the national electronic health records for follow-up. We excluded patients who underwent transplant and burns surgery, and evaluated only the index surgery for patients who had multiple surgeries during the study period. Surgeries ranged from minor day case surgeries to major surgeries. Surgical disciplines that were included were: cardiothoracic, orthopaedics, obstetrics and gynaecology, general surgery, otolaryngorhinology, hand surgery, neurosurgery, colorectal surgery, urology, plastic surgery, and oromaxillofacial surgery.

**Fig 1:**
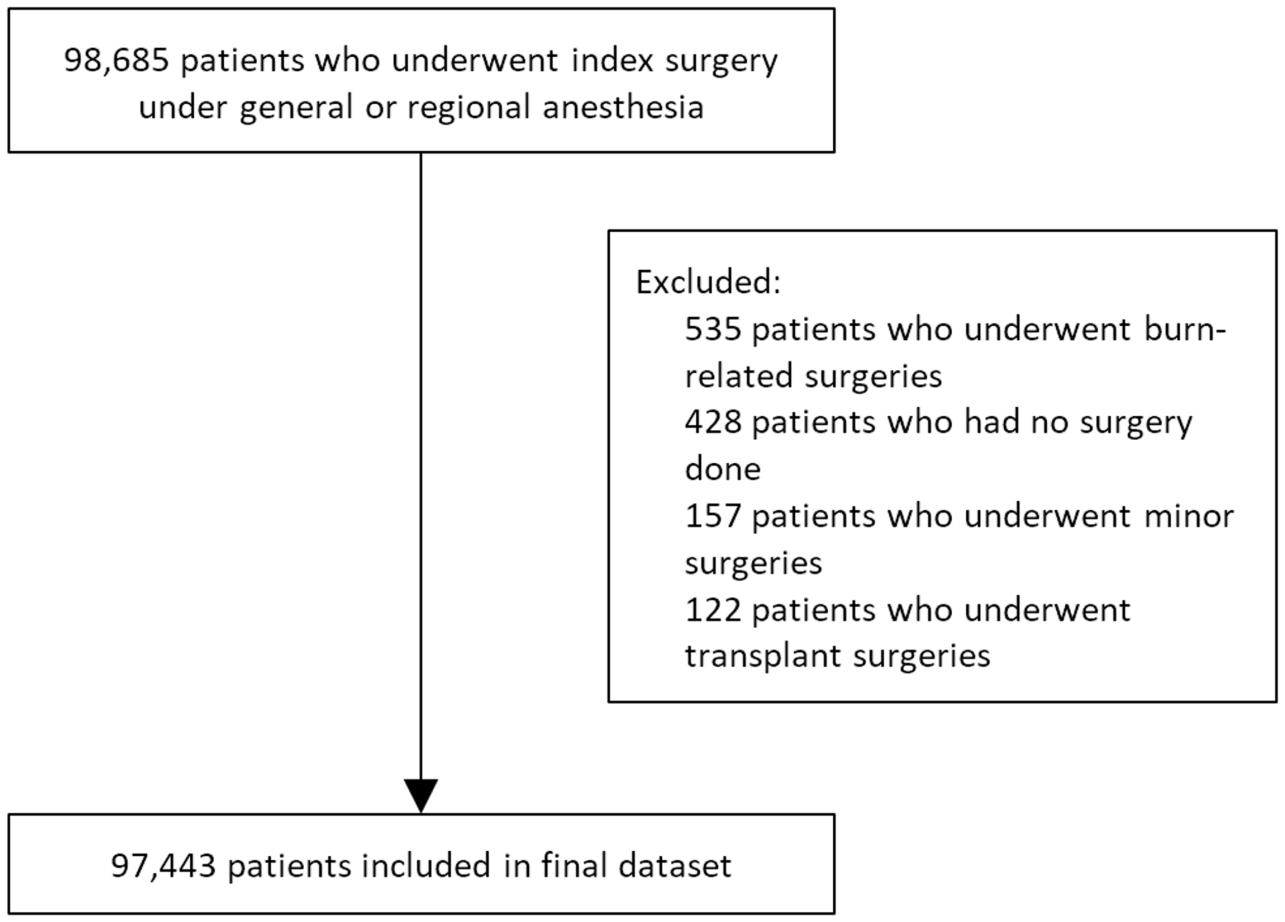
Flowchart showing study cohort derivation.

Our final dataset comprised of 97,443 patients. (Fig 1) Data collected include patient demographics, preoperative comorbidities indices such as the ASA-PS score[27], Revised Cardiac Risk Index (RCRI) score[28] and its components such as a history of previous cerebrovascular accidents (CVA), ischemic heart disease (IHD), congestive heart failure (CHF), diabetes mellitus (DM) on insulin; priority of surgery and surgical risk classification based on the 2014 ESC/ESA guidelines on non-cardiac surgery [29,30]. These information were routinely collected during the preoperative anesthesia assessment visit. Perioperative blood transfusion data was also obtained. Our missing data on preoperative hemoglobin is about 4.68%. Due to incomplete data, we analysed 57,808 patients (59.3% of the cohort) in the multivariate regression and 77,485 patients (79.5% of the cohort) in the cox regression.

### Procedures and definitions

Preoperative laboratory investigations were performed within 90 days before the surgery, up to the day of surgery but before the surgical start time. These include serum hemoglobin, red blood cell distribution width(RDW), mean corpuscular volume (MCV) and serum creatinine levels. Anemia was defined by the World Health Organization (WHO)’s gender-based classification of anemia severity.[31] Mild anemia was defined as hemoglobin concentration of 11–12.9g/dL in males and 11–11.9g/dL in females; moderate anemia was defined for both genders to be hemoglobin concentration between 8–10.9g/dL and severe anemia defined as hemoglobin concentration <8.0g/dL.

We defined perioperative blood transfusion as red blood cell (RBC) concentrate units given during the surgery and up to one month after the date of surgery. Preexisting chronic kidney disease was calculated based the estimated glomerular filtration rate (GFR) that was derived from the latest preoperative serum creatinine levels by the MDRD equation according to KDIGO guidelines.[32] RDW was reported as a coefficient of variation (percentage) of red blood cell volume with the normal reference range for RDW in this hospital laboratory to be 10.9% to 15.7%. Levels above 15.7% were defined *a priori* as high RDW. This corresponded to the 90th centile of RDW values in our study population. MCV is defined *a priori* as microcytic if < 80fL, normocytic if is between 80-100fL, and macrocytic if >100fL.

We followed up 88.7% of our patients to 1 year. 11.2% did not achieve 1 year follow up as their date of surgery was less than a year from the assessment of mortality rates. Nevertheless, the mean follow up duration was 258 days (± 64), with a minimum of 147 days.

### Statistical analysis

Statistical analysis was done in IBM SPSS Statistics v21.0. Patient data were de-identified and analyzed anonymously. Baseline characteristics of the whole cohort was determined. Proportion of categorical variables were compared using the chi-square test, while mean values of continuous variables were compared using the one-way analysis of variance (ANOVA). Multinomial regression was performed to determine patient characteristics associated with odds of mild or moderate/severe anemia over no anemia, while binomial regression was performed to determine patient characteristics that are independently associated with higher odds of high RDW (>15.7) over normal RDW (< = 15.7). In addition, cox regression analysis was done to assess the independent effect of preoperative anemia, abnormal RDW and MCV values on 1-year mortality rates. We found the interaction term between RDW, anemia and MCV to be significant, and did further subgroup analysis of the anemia categories stratified by MCV and RDW. We confirmed the proportionate hazard assumption by plotting the individual log minus log curves for each categorical variables in the cox regression.

## Results

In our cohort, 94.7% underwent non-cardiac surgeries, 5.3% underwent cardiac surgeries. The overall prevalence of WHO-defined anemia in our patient population was 27.8%—mild anemia made up 15.3%, moderate anemia 12.0% and severe anemia 0.5%. The mean RDW of our population was 13.7% (± 2.09). On the overall, normocytosis was most prevalent at 87.5%, followed by microcytosis at 11.0% and macrocytosis at 1.5%. Amongst anemic patients, normocytosis was also most prevalent at 70.7%, followed by microcytosis 26.6% and macrocytosis 2.7%. Overall one-year mortality was 3.5% - 3.5% in non-cardiac surgeries and 5.0% in cardiac surgeries. The other characteristics of our cohort are laid out in Table 1.

**Table 1:** Distribution of patient characteristics according to anemia status, RDW and MCV values.

		No anemia—N(%)	Mild Anemia—N (%)	Moderate/severe anemia—N(%)	RDW—Mean (SD)	Low MCV—N(%)	High MCV—N(%)	Normal MCV—N(%)
Age (years)	18–29	8143 (83.3%)	922 (9.4%)	712 (7.3%)	13.34 (1.77)	1236 (13.1%)	24 (0.3%)	8206 (86.7%)
	30–49	20116 (76.1%)	3268 (12.4%)	3049 (11.5%)	13.82 (2.43)	3923 (15.2%)	177 (0.7%)	21637 (84.1%)
	50–69	29883 (74.0%)	6088 (15.1%)	4416 (10.9%)	13.62 (1.91)	3366 (8.7%)	658 (1.7%)	34630 (89.6%)
	> = 70	8957 (55.0%)	3911 (24.0%)	3420 (21.0%)	13.98 (2.04)	1315 (8.3%)	510 (3.2%)	14028 (88.5%)
Gender	Male	33721 (76.3%)	6652 (15.0%)	3835 (8.7%)	13.47 (1.77)	3621 (8.5%)	729 (1.7%)	38234 (89.8%)
	Female	33378 (68.6%)	7537 (15.5%)	7762 (15.9%)	13.93 (2.31)	6219 (13.2%)	640 (1.4%)	40267 (85.4%)
Race	Chinese	48557 (73.0%)	10000 (15.0%)	7941 (11.9%)	13.65 (2.08)	5959 (9.3%)	1198 (1.9%)	57083 (88.9%)
	Malay	6162 (65.5%)	1620 (17.2%)	1623 (17.3%)	14.04 (2.20)	1503 (16.5%)	56 (0.6%)	7550 (82.9%)
	Indian	5825 (70.7%)	1343 (16.3%)	1066 (12.9%)	13.88 (1.99)	1191 (14.9%)	39 (0.5%)	6789 (84.7%)
	Others	6547 (75.0%)	1222 (14.0%)	963 (11.0%)	13.67 (2.07)	1184 (14.2%)	75 (0.9%)	7067 (84.9%)
ASA-PS	1	17076 (84.0%)	2133 (10.5)	1121 (5.5%)	13.31 (1.76)	2107 (10.6%)	96 (0.5%)	17591 (88.9%)
	2	37102 (76.1%)	7014 (14.4%)	4656 (9.5%)	13.65 (2.07)	5274 (11.1%)	640 (1.4%)	41399 (87.5%)
	3	8983 (52.1%)	3884 (22.5%)	4366 (25.3%)	14.24 (2.29)	1724 (10.6%)	503 (3.1%)	13978 (86.3%)
	4&5	917 (43.5%)	449 (21.3%)	740 (35.1%)	14.67 (2.44)	197 (9.9%)	63 (3.2%)	1736 (87.0%)
Grade of kidney disease	1	38464 (76.7%)	6626 (13.2%)	5051 (10.1%)	13.66 (2.23)	6220 (12.4%)	566 (1.1%)	43189 (86.4%)
	2	19687 (77.0%)	3704 (14.5%)	2164 (8.5%)	13.53 (1.73)	2091 (8.2%)	369 (1.5%)	22950 (90.3%)
	3	2659 (45.9%)	1597 (27.6%)	1533 (26.5%)	14.07 (1.95)	479 (8.4%)	173 (3.0%)	5063 (88.6%)
	4–5	530 (15.3%)	941 (27.1%)	2004 (57.7%)	14.87 (1.95)	244 (7.0%)	236 (6.8%)	2987 (86.2%)
Congestive Heart Failure		571 (40.9%)	376 (27.0%)	448 (32.1%)	14.83 (2.52)	131 (10.2%)	50 (3.9%)	1107 (85.9%)
Diabetes Mellitus on insulin		932 (40.4%)	577 (25.0%)	798 (34.6%)	14.10 (2.07)	272 (12.4%)^a^	44 (2.0%) ^a^	1886 (85.6%) ^a^
Priority of Surgery	Elective	55109 (74.6%)	10975 (14.9%)	7812 (10.6%)	13.69 (2.11)	7549 (10.6%)	1090 (1.5%)	62342 (87.8%)
	Emergency	11990 (63.1%)	3214 (16.9%)	3785 (19.9%)	13.78 (2.01)	2291 (12.2%)	279 (1.5%)	16159 (86.3%)
Surgery Risk	Low	34255 (76.5%)	5956 (13.3%)	4543 (10.2%)	13.51 (1.86)	4595 (10.6%)	587 (1.4%)	38292 (88.1%)
	Moderate	27129 (69.5)	6394 (16.4%)	5538 (14.2%)	13.87 (2.27)	4470 (11.6%)	624 (1.6%)	33357 (86.8%)
	High	2587 (57.8%)	912 (20.4%)	976 (21.8%)	14.26 (2.47)	484 (11.7%)	104 (2.5%)	3565 (85.8%)
One-year Mortality	Yes	2122 (34.4%)	1538 (24.9%)	2511 (40.7%)	15.3 (2.8)	409 (12.4%)	157 (4.7%)	2744 (82.9%)
	No	64977 (74.9%)	12651 (14.6%)	9086 (10.5%)	13.7 (2.0)	9431 (10.9%)	1212 (1.4%)	75757 (87.7%)

American Society of Anesthesiologists Physical Status (ASA-PS); Mean Corpuscular Volume (MCV); Red Cell distribution Width (RDW);

% displayed out of row total

^a^ p = 0.006. All other variables in the table have P values of <0.001 based on chi square testing.

**Fig 2:**
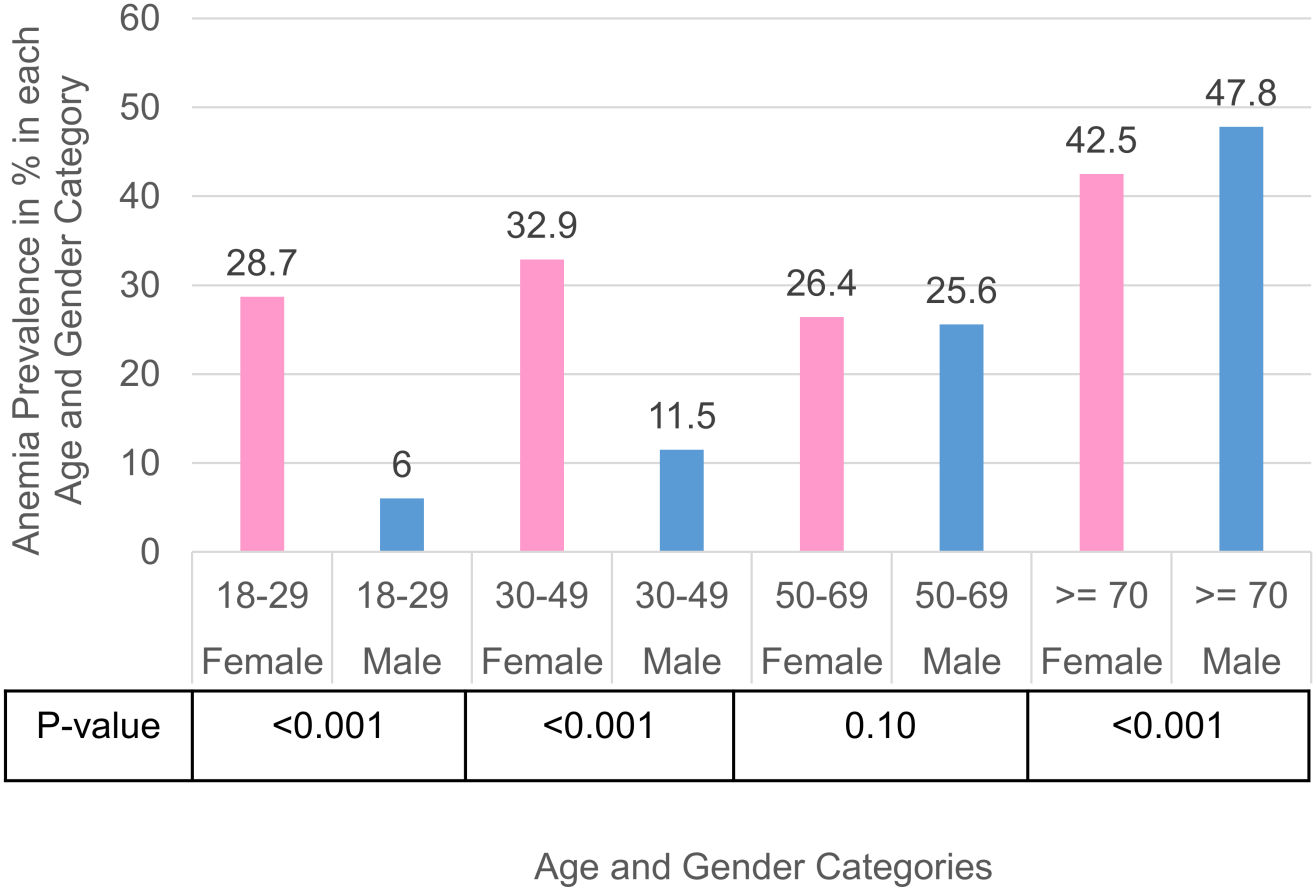
Prevalence of anemia in each age and gender groups.

**Fig 3:**
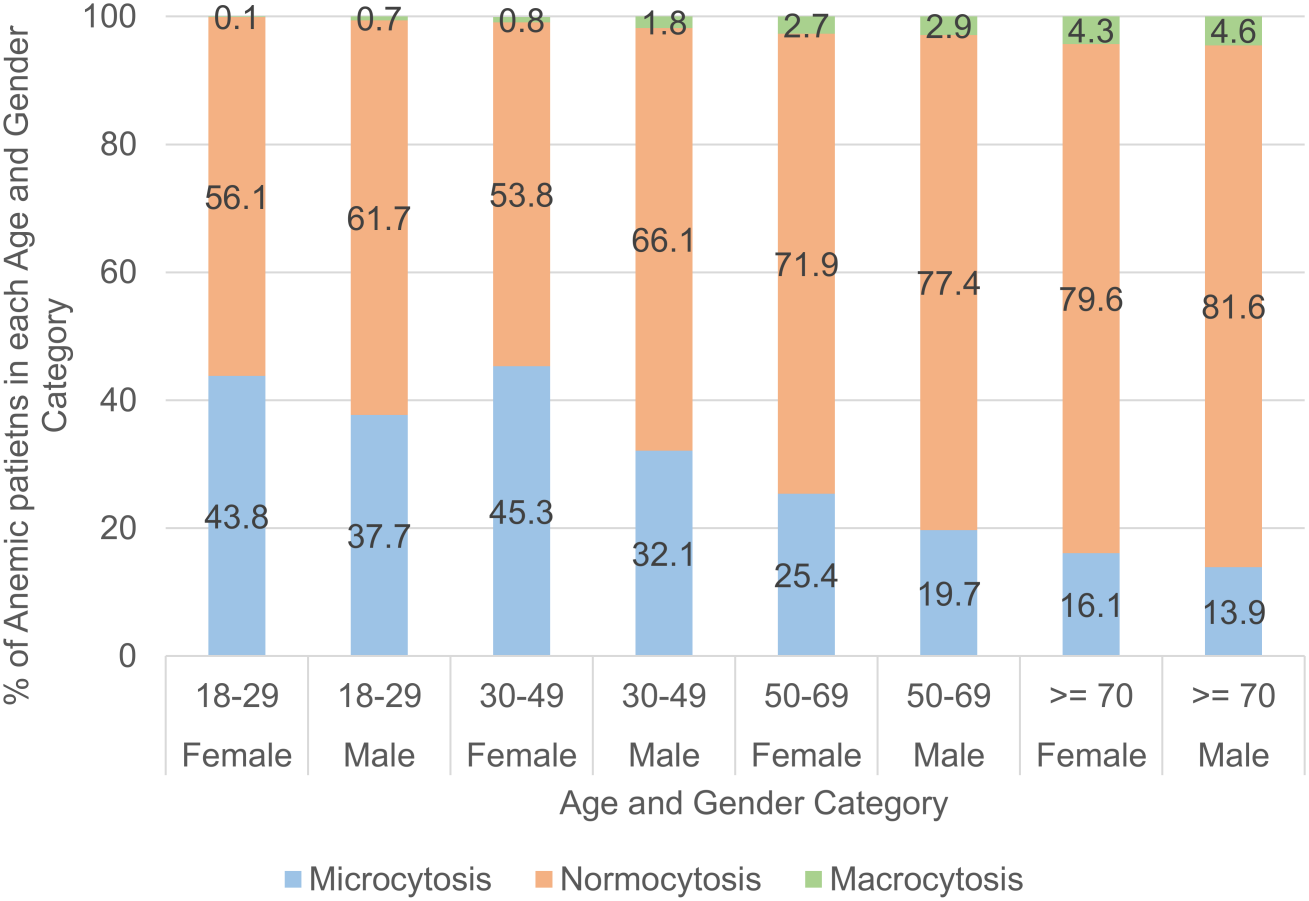
Distribution of microcytosis, normocytosis, macrocytosis in anemic patients of various age and gender groups.

The relationship between anemia prevalence and age is further illustrated in Figs 2 and 3. In Fig 2, the trend of anemia with age differs between the genders. In males, anemia prevalence increases from 6.0% at 18–29 years, to 47.8% at ages > = 70 years. However, in females, anemia prevalence is higher in reproductive ages 18–49 years; lowest at 50–69 years and rises again after. Apart from the 50–69 age group, there is a significant difference in anemia prevalence between the genders. As seen in Fig 3, in anemic patients, the proportion of microcytic anemia appears to decrease in both genders with age, while normocytosis and macrocytosis increases with age in both genders.

As shown in Table 2, the odds of both mild anemia and moderate/severe anemia compared to no anemia is highest in the oldest age group (≥ 70 years), female gender, higher ASA-PS scores (4 & 5), diabetes mellitus on insulin, congestive heart failure and grade 4 or 5 kidney disease. Patients presenting for moderate or high risk surgeries, and emergencies also have higher odds of moderate/severe anemia.

**Table 2:** Results of multinomial analysis of odds of mild or moderate/severe anemia over no anemia.

		Mild Anemia^a^		Moderate/Severe Anemia^a^	
		aOR (95% CI)	P-value	aOR (95% CI)	P-value
Age	18–29	REF		REF	
	30–49	1.55 (1.38–1.74)	<0.001	1.70 (1.49–1.94)	<0.001
	50–69	1.72 (1.54–1.94)	<0.001	1.20 (1.05–1.37)	.008
	> = 70	3.19 (2.81–3.62)	<0.001	2.35 (2.04–2.72)	<0.001
Race	Chinese	REF		REF	
	Malay	1.30 (1.2–1.41)	<0.001	1.34 (1.22–1.46)	<0.001
	Indian	1.27 (1.17–1.39)	<0.001	1.19 (1.08–1.32)	.001
	Others	1.16 (1.06–1.26)	.001	1.13 (1.02–1.25)	.018
Gender	Male	REF		REF	
	Female	1.21 (1.15–1.27)	<0.001	2.47 (2.33–2.63)	<0.001
ASA-PS	1	REF		REF	
	2	1.28 (1.19–1.38)	<0.001	1.86 (1.69–2.04)	<0.001
	3	2.14 (1.95–2.35)	<0.001	5.21 (4.67–5.80)	<0.001
	4&5	2.23 (1.80–2.76)	<0.001	7.04 (5.74–8.63)	<0.001
Diabetes Mellitus on insulin		1.62 (1.53–1.84)	<0.001	1.87 (1.64–2.13)	<0.001
Congestive Heart Failure		1.32 (1.08–1.61)	.006	1.51 (1.24–1.83)	<0.001
Grade of kidney disease	1	REF		REF	
	2	0.86 (0.82–0.91)	<0.001	0.75 (0.70–0.80)	<0.001
	3	2.01 (1.83–2.20)	<0.001	2.34 (2.12–2.59)	<0.001
	4&5	5.37 (4.64–6.21)	<0.001	11.57 (10.07–13.28)	<0.001
Surgical risk	Low	REF		REF	
	Moderate	1.19 (1.13–1.25)	<0.001	1.30 (1.23–1.38)	<0.001
	High	1.73 (1.56–1.92)	<0.001	2.07 (1.85–2.32)	<0.001
Priority of Surgery	Elective	REF		REF	
	Emergency	1.19 (1.11–1.27)	<0.001	1.69 (1.58–1.81)	<0.001

Adjusted Odds Ratio (aOR); American Society of Anesthesiologists Physical Status (ASA-PS); Mean Corpuscular Volume (MCV); Red Cell distribution Width (RDW); reference variable (REF)

^a^ Reference category in multinomial regression: no anemia

As seen in Table 3, patients with anemia and abnormal mean cell volume (MCV) are more likely to have high RDW. Indeed, the greater the degree of anemia severity, the higher the odds of elevated RDW. Mild anemia had an aOR of 3.53 (3.21–3.88) while moderate/severe anemia had an aOR of 12.37 (11.27–13.58). Strikingly, microcytosis (MCV < 80fL) is strongly associated with high RDW (aOR 17.98, 16.59–19.48) while macrocytosis is less strongly associated (aOR 1.82, 1.47–2.26).

**Table 3:** Results of multivariate analysis of odds of high RDW over normal RDW.

		aOR of high RDW over normal RDW (95% CI)	P-value
Age	18–29	REF	
	30–49	1.45 (1.24–1.70)	<0.001
	50–69	0.995 (0.85–1.17)	0.96
	> = 70	0.92 (0.77–1.10)	0.34
Race	Chinese	REF	
	Malay	0.99 (0.89–1.11)	0.91
	Indian	0.82 (0.72–0.93)	0.003
	Others	0.91 (0.79–1.03)	0.13
Gender	Male	REF	
	Female	1.04 (0.97–1.12)	0.30
ASA-PS	1	REF	
	2	1.30 (1.16–1.45)	<0.001
	3	2.46 (2.14–2.82)	<0.001
	4&5	5.24 (4.18–6.57)	0.18
Diabetes Mellitus on insulin		0.74 (0.63–0.87)	<0.001
Congestive Heart Failure		1.65 (1.34–2.02)	<0.001
Anemia	None	REF	
	Mild	3.53 (3.21–3.88)	<0.001
	Mod/Severe	12.37 (11.27–13.58)	<0.001
MCV	Normal	REF	
	Low	17.98 (16.59–19.48)	<0.001
	High	1.82 (1.47–2.26)	<0.001
Grade of kidney disease	1	REF	
	2	0.79 (0.72–0.87)	<0.001
	3	0.71 (0.61–0.82)	<0.001
	4&5	1.02 (0.88–1.18)	0.80
Surgical risk	Low	REF	
	Moderate	1.28 (1.19–1.39)	<0.001
	High	1.68 (1.46–1.93)	<0.001
Priority of Surgery	Elective	REF	
	Emergency	0.72 (0.65–0.79)	<0.001

Adjusted Odds Ratio (aOR); American Society of Anesthesiologists Physical Status (ASA-PS); Mean Corpuscular Volume (MCV); Red Cell distribution Width (RDW); reference variable (REF)

Based on Table 4, the most significant contributors to risk of one year mortality are increasing age, higher ASA scores, presence of any form of anemia, macrocytosis, high RDW, emergency surgeries and surgeries of moderate /high risks and receiving perioperative blood transfusion.

**Table 4:** Cox regression of one year mortality after surgery.

		aHR of one year mortality after surgery (95% CI)	P-value
Age	18–29	REF	
	30–49	1.57 (1.13–2.18)	0.008
	50–69	3.01 (2.19–4.13)	<0.001
	> = 70	3.92 (2.84–5.40)	<0.001
Race	Chinese	REF	
	Malay	1.03 (0.92–1.17)	0.59
	Indian	0.90 (0.78–1.05)	0.17
	Others	0.65 (0.54–0.78)	<0.001
Gender	Male	REF	
	Female	0.73 (0.67–0.79)	<0.001
ASA-PS	1	REF	
	2	3.85 (2.85–5.21)	<0.001
	3	12.08 (8.90–16.39)	<0.001
	4&5	24.36 (17.66–33.58)	<0.001
Anemia	None	REF	
	Mild	1.98 (1.77–2.21)	<0.001
	Mod/Severe	2.86 (2.56–3.20)	<0.001
MCV	Normal	REF	
	Low	0.86 (0.72–1.03)	0.10
	High	1.47 (1.19–1.83)	<0.001
High RDW		2.34 (2.12–2.58)	<0.001
Grade of kidney disease	1	REF	
	2	0.72 (0.65–0.80)	<0.001
	3	0.88 (0.78–0.99)	0.03
	4&5	1.05 (0.94–1.18)	0.40
Surgical risk	Low	REF	
	Moderate	1.33 (1.22–1.45)	<0.001
	High	1.94 (1.72–2.18)	<0.001
Priority of Surgery	Elective	REF	
	Emergency	1.62 (1.49–1.76)	<0.001
Perioperative transfusion	0 unit	REF	
	1 unit	1.41 (1.27–1.57)	<0.001
	2 or more units	1.61 (1.38–1.86)	<0.001

Adjusted Hazard Ratio (aHR); American Society of Anesthesiologists Physical Status (ASA-PS); Mean Corpuscular Volume (MCV); Red Cell distribution Width (RDW); reference variable (REF) Interaction term between anemia*MCV*RDW is significant, P-value<0.001

**Fig 4:**
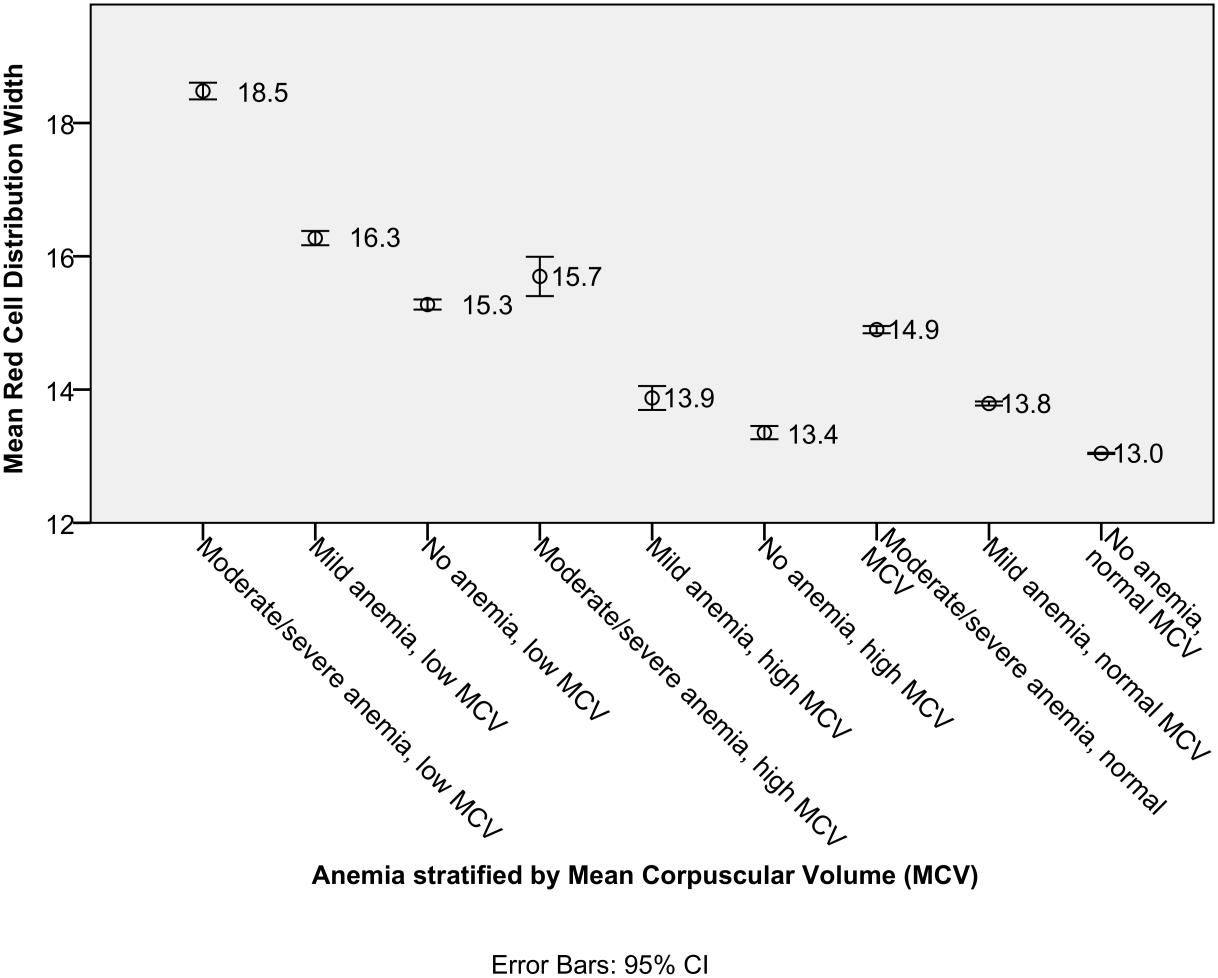
Graph showing the mean and 95% confidence intervals of red cell distribution width in various anemia / mean corpuscular volume (MCV) groups.

As seen in Fig 4, within each MCV category, the more severe the anemia, the higher the mean RDW level. Patients with moderate/severe anemia and low MCV have the highest mean RDW level of 18.5. Patients with low MCV tend to have higher mean RDW levels compared to patients with the same degree of anemia but with normal or high MCV levels.

**Fig 5:**
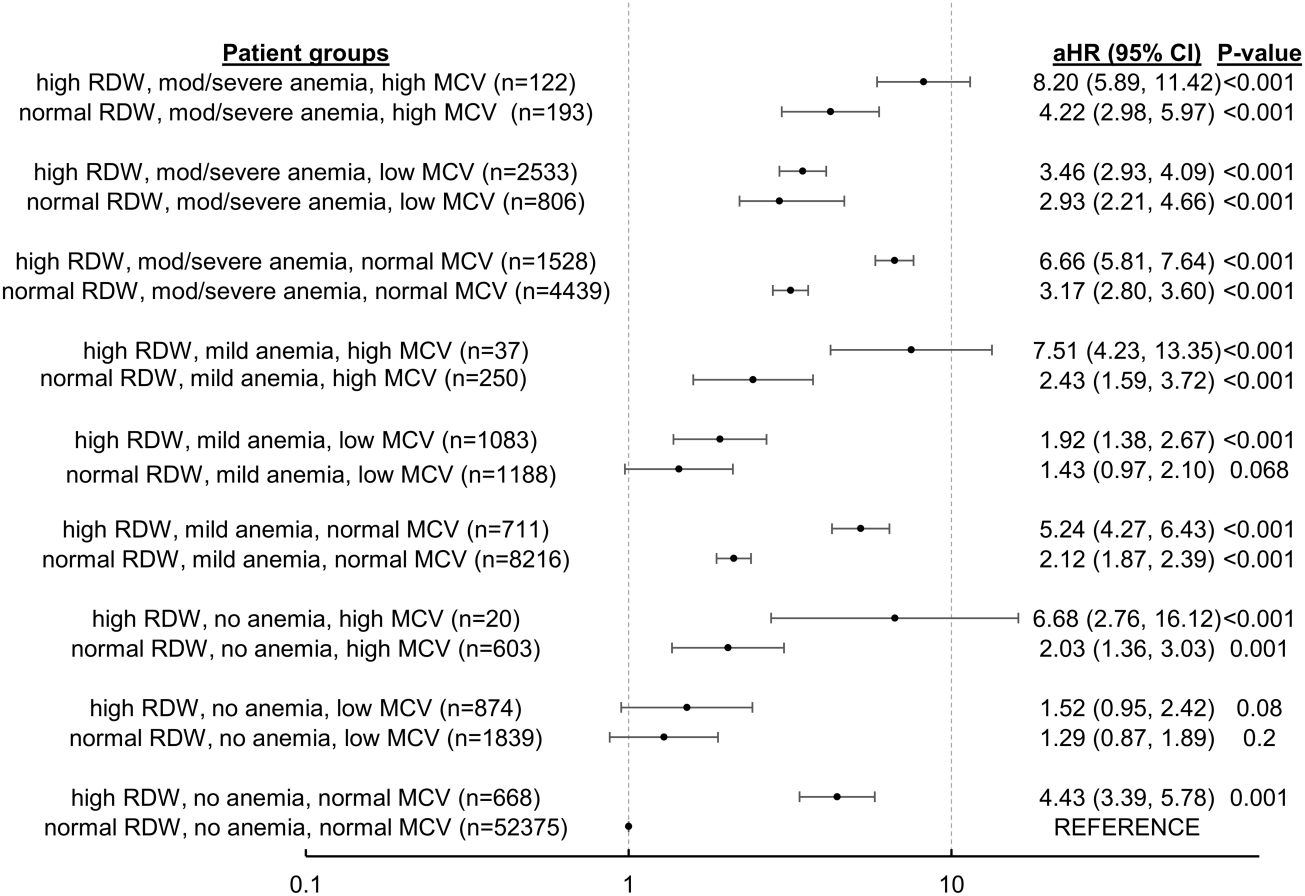
Forrest plot of aHR of 1 year mortality of anemia stratified by MCV and RDW. * adjusted for the same variables as in Table 4. Adjusted Hazard Ratio (aHR); Red Cell distribution Width (RDW); Mean Corpuscular Volume (MCV).

As the interaction term between anemia, RDW and MCV was significant in the cox regression for 1 year mortality, we repeated the cox regression with anemia stratified by RDW and MCV values, and the adjusted hazard ratio (aHR) is presented in Fig 5.

Consistently, for the same degree of anemia and type of MCV, patients with high RDW had higher aHR of mortality than those with normal RDW. This separation in mortality risk was most prominent in those with normal MCV values—patients with moderate/severe anemia, normocytosis and high RDW had an aHR of 6.66 compared to similar patients with normal RDW whose aHR was 3.17; patients with mild anemia, normocytosis and high RDW had an aHR 5.24 compared to similar patients with normal RDW whose aHR was 2.12; likewise, patients with no anemia, normocytosis and high RDW had an aHR of 4.43 compared to similar patients with normal RDW which is the reference value of 1.

Highest odds of mortality are seen in patients with macrocytosis and high RDW—patients with moderate/severe anemia had an aHR of 8.20; those with mild anemia had an aHR of 7.51, and those with no anemia had an aHR 6.68; in contrast, lowest odds of mortality are seen in patients with microcytosis with normal RDW—patients with moderate/severe anemia had an aHR of 2.93; those with mild anemia had an aHR of 1.43 and those without anemia had an aHR of 1.29.

## Discussion

Our study establishes that preoperative anemia, macrocytosis and high RDW (> 15.7%) independently increases the risk of one-year mortality after surgery.

### Anemia

The overall prevalence of preoperative anemia was 27.8% of our surgical population. The odds of moderate/severe anemia over no anemia increases with increasing age of the patient, female gender, comorbidities such as renal impairment, diabetes mellitus on insulin, congestive heart failure and higher ASA scores. Our prevalence rates are similar to that reported in other large scale studies which is between 25.3% [1], 28.7% [3] and 39.7%[2]. As we included patients undergoing minor to major, elective and emergency surgeries, our anemia prevalence differs slightly but is still within the anticipated prevalence as reported in other studies, which may have included a different assortment of surgeries in their cohort.

We found a pronounced gender difference in anemia prevalence across different age groups. Our finding corroborates well with National Health and Nutrition Examination Survey (NHANES) III study of the anemia prevalence in the civilian population in the United States[7]. In both the NHANES III study and our study, females of reproductive age (18–49 years) had a higher prevalence of anemia compared to females in the post-menopausal group (aged 50–69 years in our study, and 50-64years in the NHANES III study). This may be attributed to menstruation and childbearing. In men, the prevalence of anemia was lowest in age group 18–29 years, and rose with increasing age. Across age groups < 70 years, the prevalence of anemia was higher in women than in men. This trend reversed in patients aged 70 and above in our study, and this has also been demonstrated in other studies as well. Between ages 75 to 84 years, Salive et al[33] and Skjelbakken et al [8] estimated that 14.9% to 15.0% of men and 7.1% to 12.7% of women had WHO-defined anemia, while in the oldest age group (85 years and older), anemia was present in 29.6% to 30.7% of men and in 16.5% to 17.7% of women. Strikingly, our rates of anemia in each corresponding age and gender group seem to be almost double or triple that found in the NHANES III study. This could either be due to a different racial composition, or because our patients are pre-operative, and may have surgical conditions predisposing them to anemia.[9].

We also found that the morphology of anemia changes with age. Normocytic anemia was the predominant form of anemia in all age groups, but its proportion increased with age. The youngest age group (18–29 years) had the highest proportion of microcytic anemia compared to the older age groups, and this proportion decreased with increasing age, while macrocytosis increased with age. This suggests that the causes of anemia in the elderly and young adults are different. In the NHANES III study, one third of the older population above 65 years had anemia attributed to nutritional deficiencies, the remainder was attributed to anemia of chronic illnesses and unexplained anemia[7]. This survey also revealed that young women have higher prevalence of iron-deficiency compared to women above 50 years of age and men of the same age, which may account for the higher incidence of microcytic anemia in the female reproductive age group [34].

The population of Singapore is made up of 74.3% Chinese, 13.4% Malay, 9.1% Indians[35]. In our study, taking the Chinese race to be the reference group as it is the predominant racial group, Malays have aOR for moderate/severe anemia of 1.34 (p<0.001) while Indians have an aOR of 1.19 (p = 0.001). Racial differences in anemia prevalence is also found in the NHANES III survey, where blacks and hispanics tend to have more anemia compared to whites above 65 years of age.[7] Diet and certain inheritable genetic conditions may contribute to some of the observed racial difference in anemia prevalence in our study. Many Indians are vegetarians, and the iron [36] and Vitamin B12[37] bioavailability of a vegan diet is poor. A study of young Singaporean men registering for National Service in 1990 found that iron deficiency was the most common cause of anemia among Indians, while in Malays and Chinese, the most common cause of anemia was hemoglobinopathy, of which thalassemia was the most common[38]. In Singapore babies, alpha-thalassemia mutations was most common in Chinese (6.4%), followed by Indians (5.2%) then Malays (4.8%), while beta-thalassemia mutations were most common in Malays (6.3%), followed by Chinese (2.7%) and 0.7% in Indians[39]. With the introduction of widespread antenatal screening for thalassemia in Singapore, the incidence of babies born with thalassemia major has declined dramatically. In 2001, no babies were born with thalassemia major[40]. It is unlikely that thalassemia alone would account for the differences in prevalence of moderate/severe anemia between Malay and other races, because most of the thalassemic patients in Singapore are carriers(25.79%), have thalassemia minor (37.9%) or traits (7.43%).[40] However those with thalassemia minor or traits may have borderline hemoglobin levels which may dip further when other conditions associated with anemia sets in.

### Red cell distribution width and mean corpuscular volume

Red cell distribution width reflects the degree of anisocytosis in a patient’s circulating red blood cells. We found that the presence of anemia and abnormal mean cell volume, especially microcytosis, is strongly associated (aOR 17.98, p<0.001) with elevated RDW value (>15.7%). Surveys of the civilian population in the United States also found that mean MCV decreased from the 1st to the 4th RDW quartile [15,41]. In literature, the commonest causes of microcytosis with high RDW include iron deficiency anemia and thalassemia.[42–44] In our study, macrocytosis was also weakly associated with elevated RDW (aOR 1.82, p<0.001). Common causes of macrocytosis in literature that are associated with elevated RDW include myelodysplastic syndromes, vitamin B12 and folate deficiencies [45].

In our study, the odds of elevated RDW was higher with increasing ASA score, which suggests that patients with more severe comorbidities and consequently poorer health have are more likely to have high RDW values. However of the three comorbidities that we explored, only congestive heart failure, and not renal impairment and insulin-dependent diabetes mellitus, had independent odds of high RDW (>15.7%). Numerous studies have found elevated RDW in patients with heart failure to be associated with poorer prognosis[46–49]. Other cohort studies such as the NHANES III study in the United States [15,41], and in Taiwan[16] also found a trend of higher RDW in females and with increasing age. While patients of female gender and older age (> = 70 years) did have higher mean RDW levels (Table 1) in our study, these factors were not independent risk factors for the high RDW cutoff of >15.7% (90th centile) in our multivariate analysis.

### Impact on mortality

In our study, patients with mild anemia had an aHR of 1.98 (CI 1.77–2.21) for 1 year mortality while patients with moderate/severe anemia had an aHR of 2.86 (2.56–3.20). These values are adjusted for the effects of common influences such as perioperative blood transfusion, ASA score, age, surgical risk, emergency status and renal impairment. In addition to mortality, other studies have found that preoperative anemia also increases risks of adverse outcomes such as stroke and acute kidney injury in cardiac surgery[50] and length of hospital stay, intensive care admission and post-operative morbidities in the non-cardiac surgical population[1,3].

Preoperative anemia is a known risk factor for perioperative transfusion, which also has an independent adverse effect on postoperative outcomes [10]. In our analysis, we also corrected for the effect of transfusion thus lending strength to the assertion that the detrimental effects of anemia are independent of the effects of transfusion.

Elevated RDW is independently associated with an aHR of 2.34 (95% CI 2.12–2.58) for one year mortality in our study. This is consistent with findings in other studies, elevated RDW levels have been shown to be an independent marker for mortality after colorectal cancer surgery, coronary bypass surgery and in elderly patients who underwent fixation of hip fracture.[17–20,51] While we do not have data in our study on the cause of mortality, elevated RDW have been associated with an increased risk of cardiovascular events and all-cause mortality in patients with and without cardiovascular disease[14,16,41,52]. Elevated RDW is also associated with stroke occurrence[53] and increased all-cause mortality in patients admitted to intensive care[54–57].

From Fig 5, it is clear that the etiology of preoperative anemia based on different red cell morphology has a significant bearing on one year mortality. For the same degree of anemia and type of MCV, patients with high RDW had higher adjusted hazard ratios (aHR) of mortality than those with normal RDW. This is most strikingly shown in patients with no anemia and normocytosis, as those with high RDW have an aHR of 4.43 (p<0.001) for mortality compared to those with normal RDW levels. Also in our study, macrocytosis was found to be independently associated with increased aHR of 1.47 (p<0.001) for mortality, and in Fig 5, across all three groups of anemia, with normal or high RDW levels, patients with macrocytosis had higher aHR of mortality than those with normocytosis of microcytosis. Macrocytosis is uncommon in our study population—only 1.5% of our patients had macrocytosis, and its prevalence increases to 4.4–4.6% in the oldest age group. The causes of macrocytosis depends on the population surveyed, but common etiologies include alcoholism, vitamin B12 and folate deficiency, drug-induced, hypothyroidism, liver disease, myelodysplastic syndrome and aplastic anemia[45]; however we did not collect data in our patients that may explain their anemia.

These findings emphasize the incremental value of considering RDW together with MCV and hemoglobin levels when estimating the risks of mortality in a preoperative patient as the resulting risk estimates could be very different. Our findings are congruous with a cohort study of 36,292 elderly patients which found worse survival in patients with elevated RDW levels and macrocytosis, followed by normocytosis then microcytosis, in both anemic and non-anemic patients[58].

One attractive explanation for the some of the cases of abnormal MCV values and high RDW levels could be nutritional deficiency, as iron deficiency anemia and thalassemia are typically associated with microcytosis and high RDW [42–44], while myelodysplastic syndromes, vitamin B12 and folate deficiencies are associated with macrocytosis and elevated RDW[45] Despite these association, the link between nutritional deficiencies, RDW and mortality is not well supported in studies that do examine them. For instance in the InCHIANTI Study, NHANES III, and WHAS I, although a pooled meta-analysis of these studies found an association between RDW and mortality, this was not dramatically different in patients with and without nutritional deficiencies[59].

Inflammation has also been explored as an explanation for the association between high RDW levels and adverse outcomes, by reducing RBC survival and disrupting erythropoiesis through the effects of pro-inflammatory cytokines, leading to a more mixed population of RBC volumes in the circulation. Some studies have found a positive correlation between RDW and CRP and ESR levels[15,24].

### Strengths

Our study is the first to examine in detail the relationship between preoperative anemia, RDW and MCV levels and how they correlate with postoperative mortality across a broad spectrum of surgeries, from low-risk to high-risk surgeries, cardiac and non-cardiac surgery, and across a broad range of patient profiles, ranging from healthy young patients to older patients with multiple comorbidities. Another strength of our study was that its duration only spanned 4 years, so this reduces biases from changes in health care practices over time which may affect mortality rates.

### Limitations

As this is a retrospective cohort study, our study is not able to prove a causal relationship between RDW, anemia and mortality. Furthermore, we encountered missing data due to incompleteness of preoperative investigations, as not all patients who underwent surgery in our institution required full workup preoperatively, or may have done these investigations in a different institution that is not accessible to us. Consequently, we were only able to analyze 57,808 patients (59.3% of the cohort) in the multivariate regression and 77,485 patients (79.5% of the cohort) in the cox regression. We were fortunate that as our patient’s medical record database was synced with the National Death Registry, we were able to achieve 100% follow up with all our patients.

Another significant limitation of our study is the absence of information on the patient’s nutritional status, concomitant hematological and other non-hematological conditions that may explain the anemia, MCV and RDW levels. These information would have helped elucidate the causality between anemia, MCV, RDW and mortality. Nevertheless, the primary aim of this paper was to assess the preoperative prevalence and severity of anemia, to make screening efforts more targeted and cost effective.

Finally, our data is obtained by following up on a large cohort from a single center, which may limit the generalizability of the data to other populations locally and internationally. Nevertheless, we hope that given the large number of patients included in the study, the conclusion is robust and will encourage other population level studies to validate our results.

### Future direction

Our study contributes to the growing body of literature on the association between elevated RDW levels and mortality. While this association is clear, no standardized cutoff exists to define high RDW levels. We chose a higher level of 15.7%, which corresponds to the 90th centile in our study population, and also the cutoff level for normal limit in our laboratory. Our study also illustrates that high RDW with macrocytosis and normocytosis, seem to be associated with higher risk for mortality compared to microcytosis, for the same degree of anemia. Future prospective studies can be done to determine the etiology of anemia in preoperative patients and their association with RDW and MCV levels, by evaluating the patient’s nutritional status and other known etiologies, to assess if any reversible etiologies may be present, managed and reversed.

## Conclusion

Preoperative anemia is prevalent in patients presenting for surgery, and the odds of moderate to severe anemia is increased by older age of the patient, female gender, presence of comorbidities such as renal impairment, diabetes mellitus on insulin, congestive heart failure and higher ASA scores. Preoperative anemia, presence of congestive heart failure, higher ASA scores, macrocytosis and microcytosis are associated with elevated RDW levels. One year mortality is higher in those with any preoperative anemia, elevated RDW levels and macrocytosis. The etiology of preoperative anemia based on red cell morphology and anisocytosis has a significant bearing on one year mortality and should be routinely evaluated for better perioperative risk prognostication.
